# A Novel Metamaterial-Inspired RF-coil for Preclinical Dual-Nuclei MRI

**DOI:** 10.1038/s41598-018-27327-y

**Published:** 2018-06-15

**Authors:** Anna Hurshkainen, Anton Nikulin, Elodie Georget, Benoit Larrat, Djamel Berrahou, Ana Luisa Neves, Pierre Sabouroux, Stefan Enoch, Irina Melchakova, Pavel Belov, Stanislav Glybovski, Redha Abdeddaim

**Affiliations:** 1 Department of Nanophotonics and Metamaterials, ITMO University, 197101 St. Petersburg, Russia; 2 Aix Marseille University, CNRS, Centrale Marseille, Institut Fresnel, Marseille, France; 3 Commissariat à L’Energie Atomique et aux Energies Alternatives/Direction de la Recherche Fondamentale/Institut Joliot/Neurospin, 91191 Gif-sur-Yvette Cedex, France; 4 Université Paris Saclay, Orsay, France

## Abstract

In this paper, we propose, design and test a new dual-nuclei RF-coil inspired by wire metamaterial structures. The coil operates as a result of resonant excitation of hybridized eigenmodes in multimode flat periodic structures comprising several coupled thin metal strips. It was shown that the field distribution of the coil (i.e. penetration depth) can be controlled independently at two different Larmor frequencies by selecting a proper eigenmode in each of two mutually orthogonal periodic structures. The proposed coil requires no lumped capacitors to be tuned and matched. In order to demonstrate the performance of the new design, an experimental preclinical coil for ^19^F/^1^H imaging of small animals at 7.05T was engineered and tested on a homogeneous liquid phantom and *in-vivo*. The results demonstrate that the coil was both well tuned and matched at two Larmor frequencies and allowed image acquisition at both nuclei. In an *in-vivo* experiment, it was shown that without retuning the setup it was subsequently possible to obtain anatomical ^1^H images of a mouse under anesthesia with ^19^F images of a tiny tube filled with a fluorine-containing liquid and attached to the body of the mouse.

## Introduction

Magnetic resonance provides unique instrumentation for modern biomedical studies. In clinical and preclinical applications, radiofrequency (RF) coils play an important role of antennas exciting spins in a subject under an applied strong static magnetic field with a desired flip angle and receiving weak echo signals at the Larmor frequency. Thus, a signal-to-noise (SNR) ratio of images, strongly dependent on electromagnetic properties of RF coils, becomes particularly important if a density of an investigated nucleus in the sample is low.

In multi-nuclei studies, it is possible to retrieve additional imaging and/or spectroscopy information using the magnetic resonance of several nuclei of interest typically taking place at different Larmor frequencies for the same magnet field *B*_0_. The Larmor frequency of a nucleus is determined by its gyromagnetic ratio *γ* as *f*_L_ = *γ*·*B*_0_. The corresponding RF-coils, suitable for multi-nuclei MRI, must operate at all desirable Larmor frequencies simultaneously given that the input impedance is matched to the transceiver channel(s) and the RF-field distribution in a subject ensures maximum ${B}_{1}^{+}$ per unit power efficiency in transmission over the region of interest. The aforementioned properties also ensure a high SNR of the same coil in reception improving imaging quality and resolution of measured molecular spectra.

Preclinical studies impose additional limitations on RF-coil designs. The dedicated coils must be compatible with other equipment required for the biomedical experiment, such as excitation systems and sample beds. Another challenge comes from the electromagnetic environment of preclinical scanners, where RF-coils must operate inside an electrically narrow, shielded tunnel. Finally, RF-coils are typically very sensitive to variation of a subject’s electrical properties. In contrast to large coils (for instance, those used for clinical applications) where the noise from the subject dominates, in the so-called mid-range preclinical coils, the subject noise becomes comparable to the intrinsic noise of the coil^1^. This last fact results from power losses in the coil’s components and strongly depends on the coil’s design and quality of its materials.

RF-coils for small-animal imaging and spectroscopy at the fields 3–17 T are typically volumetric coils which have linear polarization of the RF magnetic field such as solenoids providing high SNR if the sample losses are not dominant^1,2^ or saddle coils^3^ as well as circular polarization coils such as ‘birdcages’^4,5^. The volume MR coil with the enhanced SNR was shown in^6^ where the comprehensive design with the tuning stability to the sample variations and large homogeneity of ${B}_{1}^{+}$ has been demonstrated. Surface coils implemented as planar/conformal loops^7–9^ are also used in specific applications. Most conventional loop RF-coil designs have electrically small dimensions. When excited by an RF-cable, they exhibit inductive input impedance and, therefore, need to be tuned and matched at the desired Larmor frequencies using matching circuits with multiple on-chip capacitors^1^. Smaller coil and bore dimensions require higher capacitance for tuning and matching which is accompanied by increasing losses and reducing SNR with rising Larmor frequencies. SNR can be substantially enhanced by using cryogenic technology^10^ where the internal coil losses are eliminated by introducing the coil in superconductive condition. Nevertheless, cryogenic coils are very expensive and are therefore not commonly used. Small-animal coils for scanning at two Larmor frequencies usually contain a tuning and matching circuit with at least two variable capacitors. A general dual-frequency matching strategy based on lumped capacitors has been proposed and validated in application to several different coils for the dual-nuclei ^19^F/^1^H imaging at 4.7 T^11^ (188 MHz and 200 MHz correspondingly). Lumped-element dual-frequency circuits have the advantage of providing similar RF-field patterns at both Larmor frequencies^11^. It has also been proposed^12^ that decoupled separate loops plugged into two separate channels of a 4 T MR system for scanning at ^1^H and ^23^Na (170 MHz and 45 MHz respectively) can be used. Tuning and matching of this coil at two desired Larmor frequencies is also obtained by means of variable capacitors.

In general, at least four independent parameters must be presented in the schematic to meet the tuning and matching conditions at two separate frequencies with no limitation of a quality factor. In practice, the capacitors of a circuit are controllable from outside a bore using long dielectric rotating fixtures. The capacitors introduce losses due to strong localization of an electric field which increases intrinsic noise. Consequently, the requirements to their quality factors are very strong.

In this work we have proposed, studied and experimentally validated a novel dual-nuclei coil design for preclinical imaging. The proposed coil uses resonant excitation of eigenmodes in multimode metamaterial-inspired structures of periodic metal strips, which makes it resonant at two Larmor frequencies with no lumped capacitors (in ladder-network coils^13,14^ a similar operational principle is used). The coil was tuned and matched once prior to measurements simply by adjusting its geometric parameters. Importantly, no lumped elements are required for operation of the coil. For the on-bench and MRI experiments we have manufactured the coil and tested it in ^19^F/^1^H imaging on a phantom and *in-vivo* with a small animal at 7.05 T, corresponding to the Larmor frequencies of 282.6 and 300.1 MHz with gyromagnetic constants of the nuclei: γ(^1^H) = 42.58 MHz/T, γ(^19^F) = 40.05 MHz/T.

## Results

**Figure 1:**
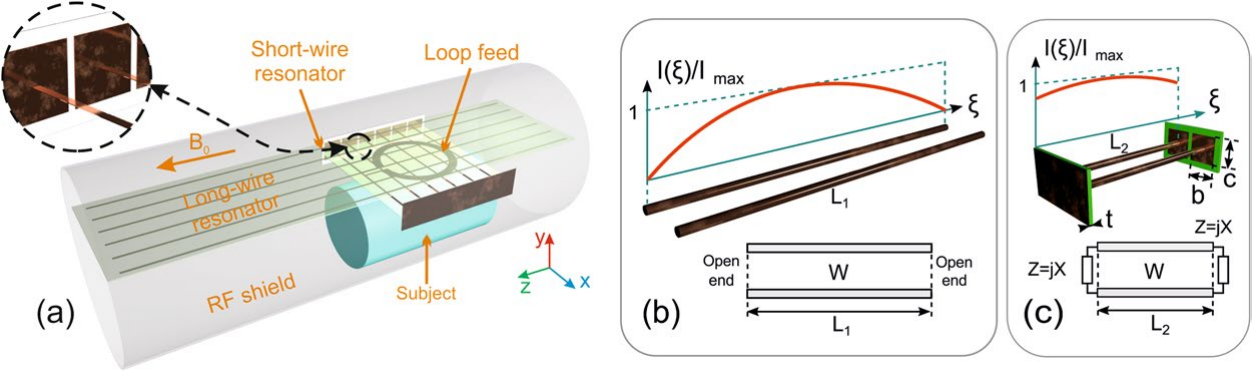
Proposed dual-nuclei RF-coil, comprising loop feed and two wire metamaterial-inspired resonators, with subject inside RF-shield of MRI (**a**); illustration of miniaturization principle for resonator of two metal wires: current distributions and equivalent circuits of resonators with two half-wavelength (**b**) and shortened (**c**) parallel wires.

The proposed dual-frequency RF-coil operates as a result of excitation of eigenmodes in two mutually orthogonal periodic arrays of parallel thin metal strips by an external non-resonant circular loop feed (in the absence of the wire resonators the resonance of the loop is well above 300 MHz) connected to a single coax cable. These two wire metamaterial-inspired resonators are referred to as the long-wire resonator (with strips parallel to the direction *z* of the static field *B*_0_ of the magnet) and the short-wire resonator (with transverse strips with respect to *B*_0_). Excitation of eigenmodes is possible owing to inductive coupling of the loop feed to both resonators. The coil geometry with a subject inside a preclinical MRI bore is depicted in Fig. 1(a). The long-wire resonator consists of identical nearly half-wavelength at 300.1 MHz strips, while the short-wire resonator for 282.6 MHz is based on shortened, but capacitively loaded, strips which fit preclinical bore dimensions. The capacitive loads connecting the adjacent strips of the short-wire resonator are rectangular copper patches of the dimensions *b* × *c* printed on a common grounded dielectric substrate of the thickness *t*. Such capacitive loading presents at both ends of the shortened strips. The patches loading the strips of the short-wire resonator are shown in the inset of Fig. 1(a) while their connection to two adjacent strips is illustrated in Fig. 1(c).

### Eigenmodes of wire metamaterial-inspired resonators

In order to demonstrate the RF-field patterns created separately by the long-wire and the short-wire metamaterial-inspired resonators of the coil at their resonant frequencies, we performed a numerical study of their eigenmodes. This resulted in two sets of resonant frequencies and two sets of RF magnetic field distributions. These electromagnetic properties are useful to describe the physical principles behind the operation of the proposed coil under realistic MRI conditions. Instead of one electric-dipole-mode resonance of a single wire, in the array of *N* half-wavelength wires (the long-wire resonator) due to the inter-wire coupling, one can excite *N* = 6 eigenmodes with different resonant frequencies (the so-called hybridization effect). Each eigenmode has its individual RF-field pattern created by a unique combination of currents flowing in positive or negative directions along the strips. Despite the fact that all currents related to a certain eigenmode have different magnitudes and phases (0 or *π* in the lossless case), their distribution along each strip is similar and cosinusoidal (like at the resonance of a single wire).

**Figure 2:**
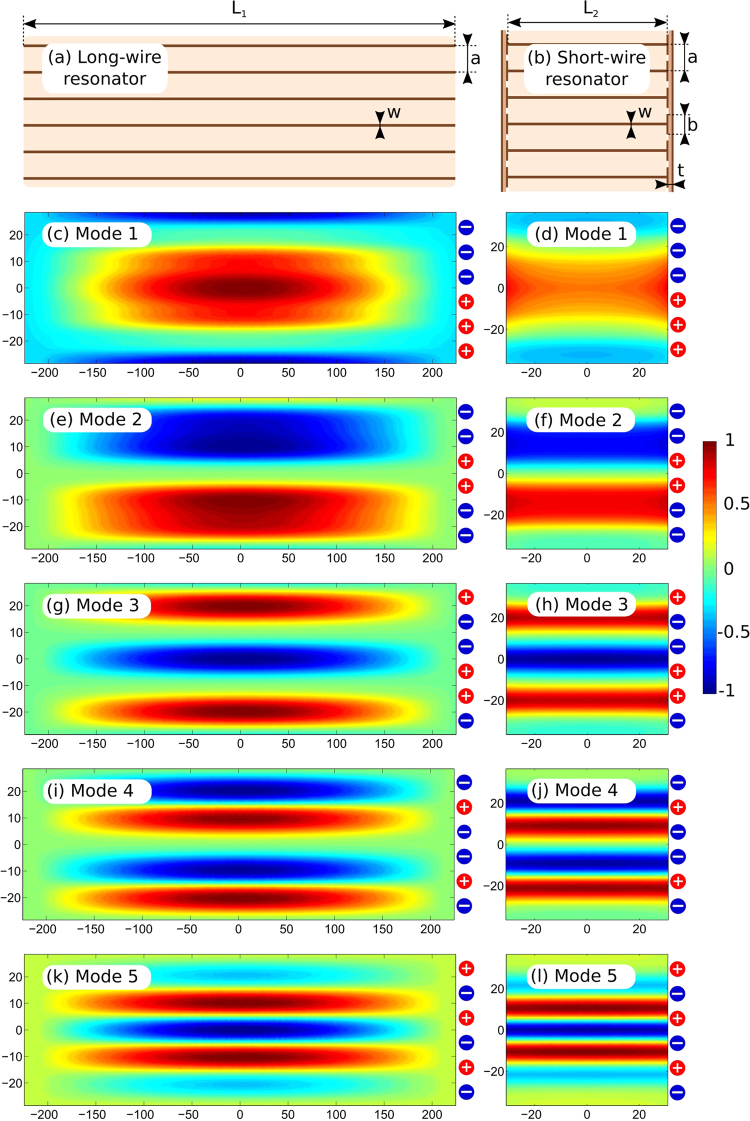
Geometry and eigenmode H-field patterns (normal component with respect to plane of strips), in a.u.: long-wire resonator (**a**,**c**,**e**,**g**,**i**,**k**) and short-wire resonator (**b**,**d**,**f**,**h**,**j**,**l**).

The numerically calculated resonant frequencies of the studied modes are listed in Table 1. In Fig. 2(c,e,g,i,k), the calculated normalized distributions of the *H*_*y*_ field component (normal to the plane of strips) are presented for five of the six available eigenmodes of the long-wire resonator suitable for preclinical MRI applications. Figure 2(a) illustrates the geometric parameters of the resonator. The field is shown in the plane parallel to the resonator, 5 mm away from the plane of strips.

**Table 1:** Orders and simulated resonant frequencies of eigenmodes.

Long-wire resonator		Short-wire resonator	
Mode order	Frequency, MHz	Mode order	Frequency, MHz
1	322.4	1	264.4
2	316.3	2	341.2
3	309.9	3	379.3
4	305.3	4	396.3
5	302.5	5	408.6

**Figure 3:**
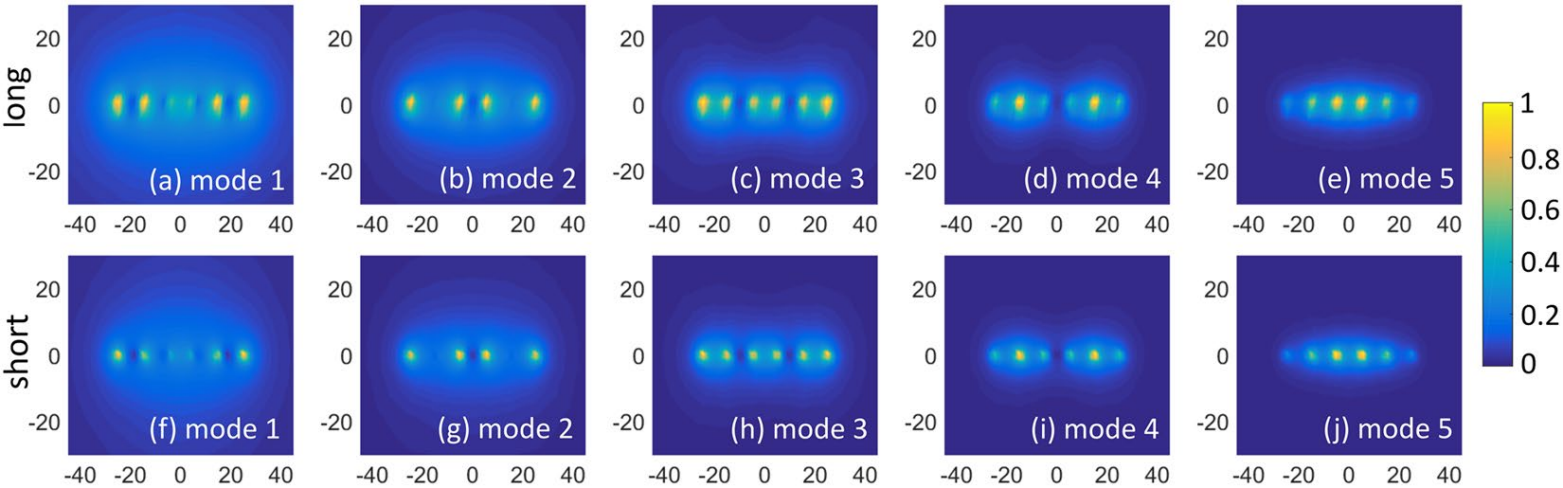
Eigenmode transverse plane H-field patterns, in a.u.: long-wire resonator (**a**–**e**) and short-wire resonator (**f**–**j**).

**Figure 4:**
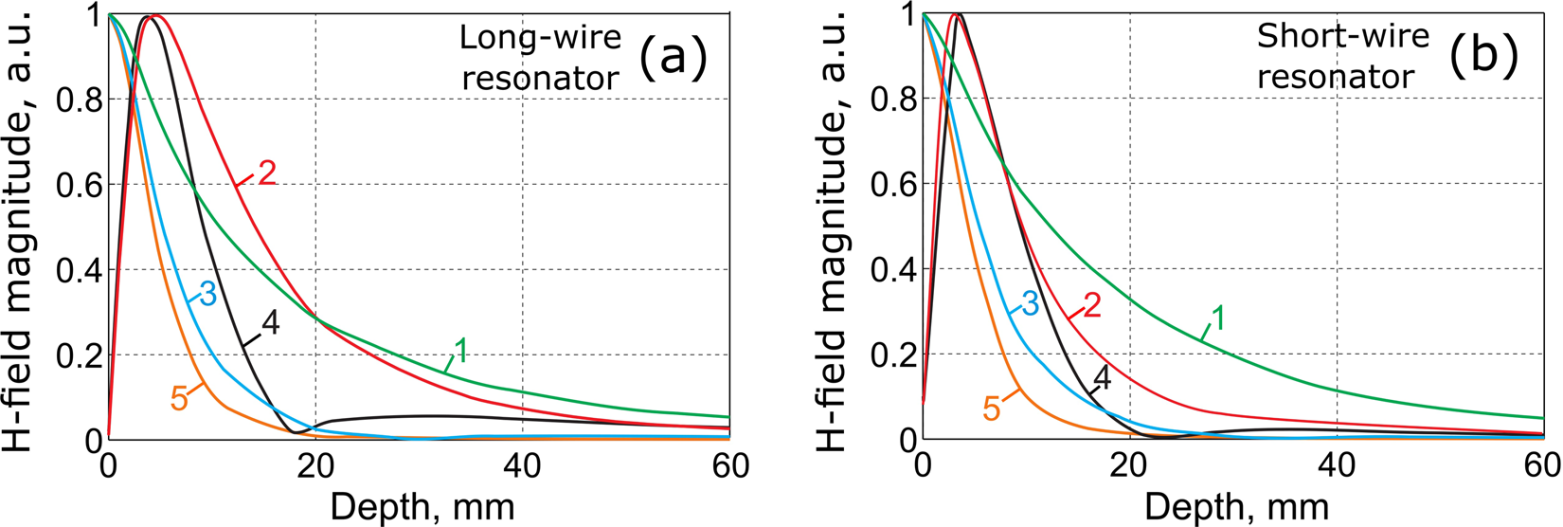
Normalized magnetic field magnitude of eigenmodes of the orders 1–5 depending on distance away from plane of strips: (**a**) long-wire resonator; (**b**) short-wire resonator.

Similar results, calculated for the resonator composed of *N* = 6 shortened strips paired by connection through structural capacities (the short-wire resonator), are presented in the right column of Fig. 2. The short-wire resonator also supports six different eigenmodes, five of which are depicted in Fig. 2(d,f,h,j,l). Figure 2(b) shows the geometric properties of the short-wire resonator. Transverse plane eigenmode H-field patterns are depicted in Fig. 3 both for the long-wire (a–e) and short-wire (f–j) resonators. In Fig. 4, the dependence of the normalized H-field magnitude as a function of the distance away from the plane of strips (the depth) is illustrated for the long-wire resonator (a) and for the short-wire resonator (b). The observation point in each case is located in front of the center of the resonator.

### Design and simulation of RF-coil

The proposed dual-nuclei RF-coil as a whole combines the long-wire and the short-wire resonators studied in the previous subsection into the same design. Strips of the two resonators are positioned in two parallel planes as shown in Fig. 1(a). The third parallel plane contains a feeding non-resonant circular loop which is inductively coupled to both resonators and is connected by a single coaxial cable to the transceiver at both Larmor frequencies. The main tuning parameters are the lengths *L*_1_ and *L*_2_ of strips, while the disposition of the loop feed with respect to the resonators is mostly responsible for matching. In the experimental antenna described in this work, these geometric properties were at once chosen and fixed for the specific sample. The sensitivity of the coil with respect to the sample variation was numerically studied by varying the sample size. In principle, the lengths of wires and the feed position could be made mechanically adjustable for compensation of significant subject variations, e.g. by using telescopic extendable tubes or worm screws.

**Figure 5:**
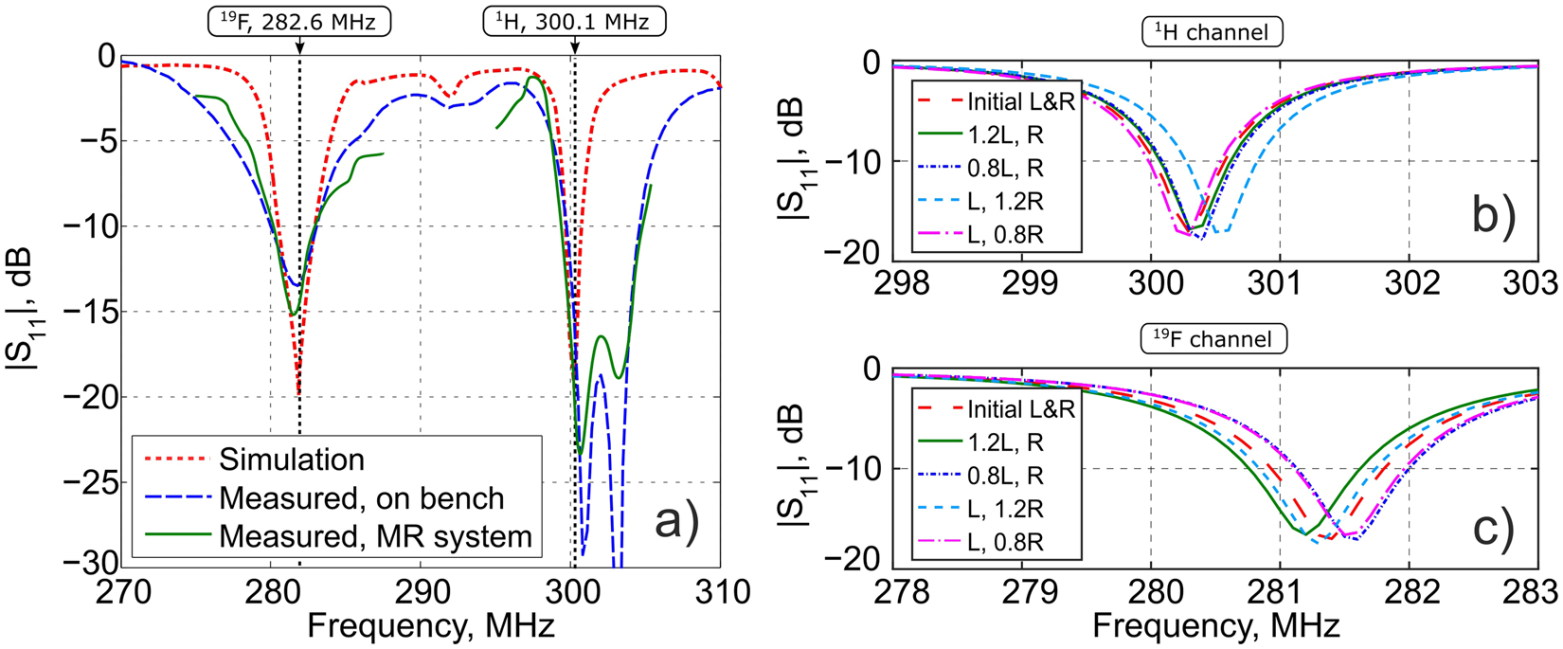
Simulated and measured values of reflection coefficient *S*_11_ of proposed coil for ^1^H/^19^F imaging vs. frequency (**a**); simulated *S*_11_ vs. frequency of proposed coil with variable phantom size for ^1^H channel (**b**) and ^19^F channel (**c**).

Selection of a proper eigenmode at each Larmor frequency allows for control of the penetration depth. We performed a full-wave numerical simulation of the assembled coil in the presence of a phantom (the equivalent of a scanned subject) and the MRI tunnel. In order to demonstrate that the desired eigenmodes can be selected and adopted for operation of our coil at the given nuclei, we tuned Mode 3 of the long-wire resonator (see Fig. 2(g)) to the Larmor frequency of ^1^H and Mode 1 of the short-wire resonator (see Fig. 2(d)) to the Larmor frequency of ^19^F. Due to the symmetry of their H-field distributions, both of these modes can be coupled to the circular loop located over the center of both resonators. Figure 5(a) shows the simulated reflection coefficient (red dotted curve) from the feeding point of the RF-coil in the split of the loop with respect to 50 Ohm cable impedance. On the phase of numerical simulation, we also analyzed the proposed coil’s sensitivity to subject variation. In particular, *S*_11_ of the coil around both Larmor frequencies was calculated with parametrically swept dimensions of the cylindrical phantom (keeping the same phantom material properties). In the calculations, three values of the phantom radius: 0.8*R*, *R* and 1.2*R*, as well as three values of the phantom length 0.8*L*, *L* and 1.2*L* were considered, where *R* and *L* are the original parameters of the numerically optimized design. In Fig. 5(b,c) the calculated *S*_11_ vs. frequency plots are compared.

As can be observed from the loading sensitivity study results, once the coil is tuned and matched by adjusting geometrical parameters of its structure (wire lengths and the loop-to-resonator gaps), the *S*_11_ stays stable enough when the phantom dimensions vary by ±20%. Therefore, the coil may be used without additional tuning and matching prior to each measurement when samples of the same type are investigated (e.g. mice of a similar kind). It was noted that when replacing a liquid cylindrical phantom by a living mouse, the coil had to be tuned and matched because two such different samples caused a larger loading variation than that caused by changing to a sample of the same type. This was done by adjusting the lengths of printed wires of the PCBs and by changing the gaps between the loop and the two resonators.

**Figure 6:**
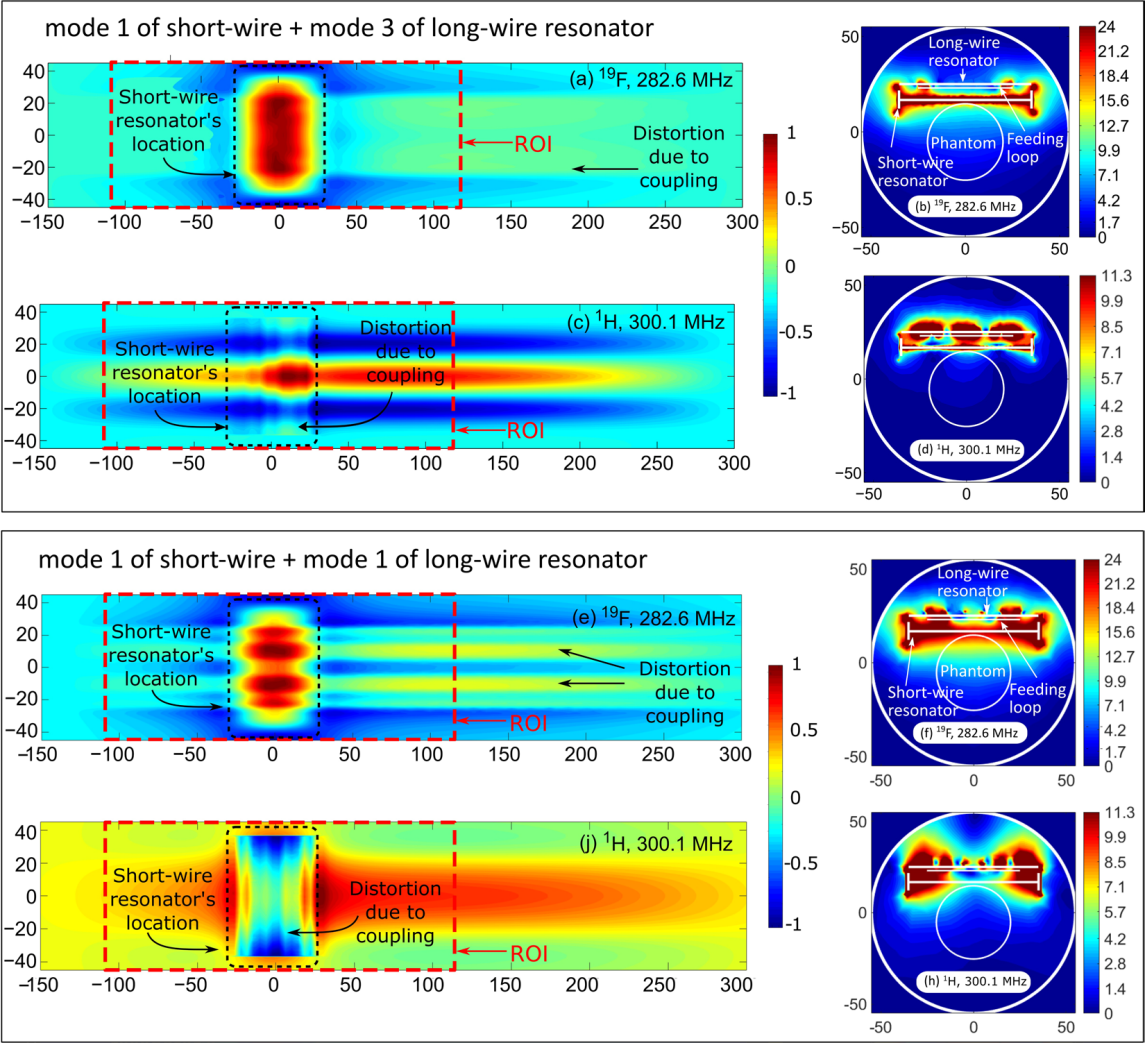
Simulated normal magnetic field component (a.u) in vicinity of RF-coil, operating using modes 1,3 (**a**,**c**) and modes 1,1 (**e**,**j**) of short and long wire resonator respectively: 282.6 MHz (**a**,**e**) and 300.1 MHz (**c**,**j**); simulated distributions of $|{B}_{1}^{+}|$ for 0.5 W accepted power, $\mu {\rm{T}}/\sqrt{{\rm{W}}}$, in central transverse plane of RF-coil operating using modes 1,3 (**b**,**d**) and modes 1,1 (**f**,**h**) of short and long wire resonator respectively: 282.6 MHz (**b**,**f**) and 300.1 MHz (**d**,**h**).

The calculated normal magnetic field component distributions created by the RF-coil are shown for ^19^F and ^1^H Larmor frequencies in Fig. 6(a) and (c) respectively. Both observation planes were chosen 5 mm away from the planes of the resonating strips. Figure 6(b,d) presents the distributions of the right-handed circularly polarized component ${B}_{1}^{+}$ at ^19^F and ^1^H Larmor frequencies respectively, which is responsible for the excitation of spins. ${B}_{1}^{+}$ is plotted in the central transverse plane of the MRI bore and the field values correspond to the accepted power of 0.5 W.

To demonstrate that another combination of eigenmodes can be selected for the same application, the other wire lengths and gaps between the loop and the wire resonators were numerically determined. As a result, it was shown that in both resonators Mode 1 can be excited, which is observed in Fig. 6(e,j). This combination provides the highest penetration of fields at both Larmor frequencies (compare Fig. 6(d) for Mode 3 and Fig. 6(h) for Mode 1 of the long-wire resonator). However, compared to the previous combination where the different modes were selected for the two resonators, similar modes cause stronger coupling between the resonators. This results in higher distortion of the field pattern depicted in Fig. 6(e,j) as compared to the corresponding eigenmode patterns (see Fig. 2(c,d)).

### MRI experiments on phantom and *in-vivo*

**Figure 7:**
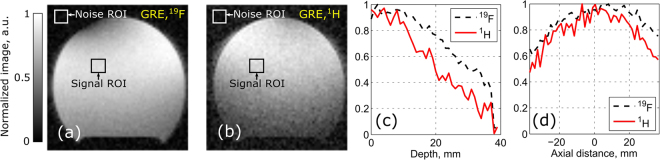
Reconstructed images acquired with Bruker PharmaScan 7 T of cylindrical liquid phantom (mixture of 60% 2-2-trifluorethanol and 40% water) with gradient echo sequence: ^19^F (**a**); ^1^H (**b**); normalized image profiles vs. depth to phantom in central transverse plane (**c**); normalized image profiles vs. axial distance along central axis of phantom (20 mm depth) (**d**).

In order to experimentally test the proposed coil, two measurements under realistic MRI conditions were performed. In the first one, we used a homogeneous liquid phantom as a scanned subject to measure the S-parameters of the manufactured coil and acquire MRI images at the two nuclei of interest. In the second (*in-vivo*) experiment, we used a mouse under anesthesia for imaging at ^1^H and a syringe with a mixture of 60% 2-2-trifluoroethanol and 40% water attached to the mouse body for imaging at ^19^F. The images of the cylindrical homogeneous phantom obtained for ^19^F and ^1^H without replacing the coil and the phantom are presented in Fig. 7. The measured SNR was calculated from the images as the ratio between the signal average and the noise standard deviation picked up in the corresponding ROI shown in Fig. 7(a,b). The distances from each of the resonators to the ROI that was chosen for SNR estimation were 18 mm for the short-wire and 27 mm for the long-wire resonator. For the hydrogen, the SNR in the ROI was 39, while for the fluorine the SNR was 63. Additionally, the decay of the image signal (in normalized image levels) at the two Larmor frequencies is illustrated in Fig. 7(c,d). The signal, as the function of the depth in the phantom in the central axial plane, is given in Fig. 7(c), while the signal depending on the axial distance along the central axis of the phantom (20 mm depth), is presented in Fig. 7(d).

**Figure 8:**
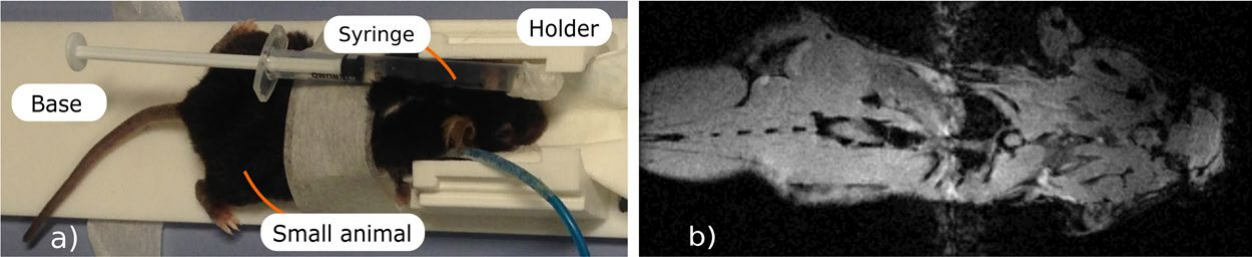
*In-vivo* setup including mouse under anesthesia and syringe containing fluorine compound (**a**) and anatomic gradient echo coronal-plane image of mouse acquired using the manufactured dual-nuclei coil (**b**).

**Figure 9:**
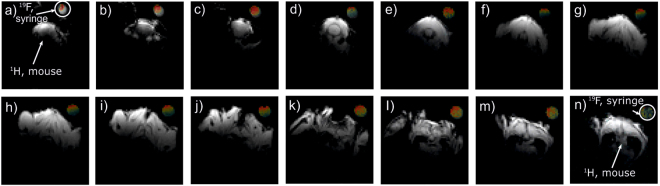
*In-vivo* anatomic gradient echo axial-plane images of mouse and ^19^F images of syringe with a mixture of 60% 2-2-trifluoroethanol and 40% water attached to mouse body in fourteen different slices acquired using proposed dual-nuclei coil.

The goal of the *in-vivo* trials with the proposed coil was to demonstrate that the latter was capable of dual-nuclei imaging without replacing and retuning the setup. In the corresponding setup, a small syringe containing a fluorine compound was attached to a mouse under anesthesia, as shown in Fig. 8(a). In Fig. 9, images for the fourteen adjacent transverse slices are displayed in a *gray* color scale showing the anatomy of the mouse body as well as the water inside the syringe. The corresponding images acquired at ^19^F are overlaid with the ^1^H images and mapped with a *jet* color scale. As expected, only the syringe filled with the fluorine compound gave any signal.

An example of a coronal ^1^H image of the whole mouse body is presented in Fig. 8(b). No fluorine is present in this plane covering only the mouse body.

## Discussion

An array of the given number *N* of half-wavelength wires supports *N* eigenmodes with different resonant frequencies. These resonances result from hybridization of the electric-dipole mode resonance of a single half-wavelength wire. This hybridization effect is due to the interaction between identical coupled resonant elements of an array. The hybridization effect in a periodic wire structure may lead to novel designs of RF-coils for MRI. Thus, the excitation of hybridized eigenmodes in arrays of coaxial resonators has been used in TEM-coils^15^. By exciting such modes in resonant metasurfaces it is possible to locally improve SNR of an external RF-coil in 1.5 T MRI. It has recently been shown^16^ that low-profile wire metamaterial-inspired resonators filled with water can serve as artificial resonant substrate locally increasing SNR of body coils. It has also been proposed that the hybridized eigenmodes excited in a system of four *λ*/2-wires could be used in a novel volumetric preclinical RF-coil for 7 T^17^.

Among the hybridized eigenmodes of the long-wire and the short-wire resonators, there is one with all the strip electric currents flowing in-phase. This mode has a similar RF-field as the dipole mode of a single resonant wire and a huge radiation leakage loss. In fact, this radiation efficiency is useful for body imaging at 7 T to extend the ${B}_{1}^{+}$ penetration and improve transmit capabilities. It is necessary for all other eigenmodes to have some out-of-phase currents pairs. A pair of currents with opposite phases produces a magnetic field localization nearby, which can provide an appropriate filling factor of a sample with dimensions comparable to the distance between the currents of the pair. In other words, for higher-order modes the H-field decays faster as function of the distance from the resonator in the normal direction. This is the common property of both of the resonators of periodic strips under consideration: the long-wire and the short-wire resonators.

For the long-wire resonator (left column in Fig. 2), all the modes have a cosine-like field distribution of the normal magnetic field component in the longitudinal direction (along the strips). At the same time, the distribution has a standing-wave shape with between one and five lobes in the transverse direction with respect to the strips. The number of lobes is related to the order of the mode. Note that in Fig. 2, the eigenmodes are sorted according to the number of standing-wave lobes (the order of the standing-wave resonance). The positive (0) or negative (*π*) phases of strip currents producing the mode patterns are indicated in each pattern by ‘+’ or ‘−’ symbols to the right of the field color plots. It can be seen that the most homogeneous field pattern (Mode 1, Fig. 2(c)) corresponds to the highest resonant frequency. In contrast, Mode 5 having five standing-wave lobes, resonates at the lowest frequency. All the simulated resonant frequencies for modes 1–5 are compared for the two resonators in Table 1.

The modes calculated for the short-wire resonator with resonant frequencies approximately in the same range of around 300 MHz (see Table 1), are depicted in the right column in Fig. 2. It can be observed that the magnetic field distributions are similar to that of the long-wire resonator, especially in terms of the standing-wave profile in the direction orthogonal to the strips. However, the field in the direction of strips is almost constant due to the presence of structural capacitive loads at the ends of the strips. In fact, for the short-wire resonator, this current profile along the strips is given by the same cosine-like function as for the long-wire resonator. The difference is that this profile is cropped by the electrically small strip lengths. The latter is illustrated by the current distributions in Fig. 1(b,c). The other difference of the short-wire resonator is the inverse order of resonant frequencies with respect to the long-wire resonator. This inverse order of modes can be explained by much higher contribution of the capacitive coupling between adjacent strips for the short-wire resonator than for the long-wire one.

In the two resonators under discussion, the mode with the most homogeneous pattern of the normal H-field component is Mode 1 (Fig. 2(c,d)). From Figs. 3 and 4, one can see that this mode has the slowest field decay as the observation point goes away from the plane of strips. It has the lowest resonance frequency of 264.4 MHz for the short-wire resonator, while it has the highest resonance frequency of 322.4 MHz for the long-wire resonator. It can be observed that the field decays faster for higher numbers of lobes. For RF-coil application, one can expect that the modes with better in-plane homogeneity of the normal H-field component show better penetration into a subject and higher ${B}_{1}^{+}$ per power efficiency at a depth. It should be noted that odd and even modes must be compared separately, as even modes have zero H-field at the center of the resonator. The highest penetration depth can be expected from Mode 1 (Fig. 2(c,d)). The distribution of electric current phases of this mode provides the field in the vicinity of the resonator is similar to the field of a long surface loop coil. If one aims to localize the field pattern in a subject’s surface layer, the mode with the most inhomogeneous normal magnetic field and current phases should be excited in the resonator (namely Mode 5, Fig. 2(k,l)). This mode is equivalent to a set of five narrow and long loop surface coils, where each loop is one period in width and the neighboring loops are excited out-of-phase, resulting in low field penetration.

In both long-wire and short-wire resonators, it is possible to excite a variety of eigenmodes, which differ by their resonant frequencies and field penetration depths. If the two resonators are combined in the same RF-coil, one can manage its field penetration into a subject at two different Larmor frequencies by selecting appropriate resonant eigenmodes (one selected mode from each of the two resonators). The two different implementations, i.e. the long and the short resonator, were used to demonstrate that the multi-wire resonator can be properly designed for a given length. If there is no capacitive loading, it is possible to use a half-wave length, but if one aims to make the wires shorter, a capacitive loading must be used according to the given theory. Significantly, the multimode principle holds in both cases. Two completely different lengths and the perpendicular positions of the combined resonators give minimal parasitic inductive coupling, which makes the coil easier in terms of individual tuning and matching at two Larmor frequencies.

For a dual-nuclei MRI, one needs to operate at two different Larmor frequencies, possibly with the same or different field patterns depending on the application. In the aforementioned resonators, it could be possible to use two of N eigenmodes exciting them at two different Larmor frequencies. However, for neither the long-wire nor the short-wire resonator, the resonant frequencies of two different eigenmodes cannot be tuned independently. Therefore, in the proposed coil, we have combined the two resonators into the same design by using just one eigenmode from each of them. Therefore, in the proposed design, the short-wire resonator is responsible for ^19^F, while the long-wire resonator is responsible for ^1^H. The small overlap area of the two resonators in comparison to the area of the long-wire resonator minimizes their mutual coupling. On the other hand, since the strips of the short-wire resonator are arranged perpendicularly to the *B*_0_ direction and the typical bore diameter of a 7 T preclinical bore is only 90 mm, the resonator must be considerably shortened in comparison to the half-wavelength. This can be achieved by capacitive connection in each pair of strips^18^ as shown in Fig. 1(c). This self-resonant geometry, comprising periodic metal wires and capacitive patches at their ends, was inspired by mushroom high-impedance surfaces^19^ and realized by connecting each printed shortened strip of the resonator end to a rectangular copper patch of the side lengths b = 9 and c = 9.5 mm. All the patches from both ends of the strips were printed on two separate Rogers 4003 C grounded substrates of the thickness t = 0.508 mm (see Figs 1(c) and 2(b)).

The idea of miniaturization by connecting adjacent strips through capacitive loads can be understood from the following example of the resonator with only two strips. The long-wire resonator with two strips of the width *w* and the separation *a*, acts as a TEM-transmission-line segment with two open ends. Even if the strips are printed on a thin dielectric substrate, the initial resonant length of this resonator is approximately equal to *L*_1_ = *λ*/2, where *λ* is the wavelength in free space (see Fig. 1(b)). In contrast, in the short-wire resonator, the parallel strips are connected at both ends to each other through two structural capacities, each one realized as a rectangular patch over a grounded substrate. The resonator, together with its equivalent circuit, are shown in Fig. 1(c). From the circuit, it is possible to derive the formula for the resonant length *L*_2_ shortened due to the capacitive loads:

1 $${L}_{2}=\frac{\lambda }{2\pi }(\arctan \frac{2WX}{{X}^{2}-{W}^{2}}+\pi ),-\,\,\infty  < X < -\,\,W;$$

2 $${L}_{2}=\frac{\lambda }{2\pi }\arctan \frac{2WX}{{X}^{2}-{W}^{2}},-\,\,W < X < 0;$$

The structural load reactance *X* can be calculated from a series connection of two similar capacities *C*_patch_ between a patch and a ground plane:

3 $$X=-\,\frac{2}{\omega {C}_{{\rm{patch}}}},$$

where *ω* = 2*πf* is the angular frequency and *C*_patch_ is given by

4 $${C}_{{\rm{patch}}}=\frac{{\varepsilon }_{r}{\varepsilon }_{0}b\cdot c}{t}.$$

The formulas (1-2) can be used for the estimation of the miniaturization factor due to capacitive loading. In other words, one can calculate the ratio *L*_1_/*L*_2_ for the given geometric parameters of patches and strips. For the above considered geometric parameters *a*, *b*, *c* and *w* at 300 MHz using the analytical formula for the wave impedance W of an edge-coupled strip line^20^, one can obtain *L*_1_/*L*_2_ = 4.3. However, in the real resonator configuration with six strips, the above formulas give only an approximation of the lengths of shortened strips. The precise values of *L*_1_ and *L*_2_ for tuning at the given Larmor frequencies depend on the orders of selected eigenmodes and can be accurately worked out numerically. The specific numerically determined length of strips of the short-wire resonator was *L*_2_ = 72 mm, while the length of the long-wire resonator was *L*_1_ = 434 mm.

The whole proposed coil (see Fig. 1(a)) has been numerically calculated in the realistic MRI setup (with a phantom and the RF-shield) to demonstrate its dual-frequency operation. For the two Larmor frequencies, corresponding to the nuclei of interest ^19^*F* and ^1^*H*, we decided to select the following modes: Mode 1 of the short-wire resonator and Mode 3 of the long-wire resonator respectively. This choice was made to demonstrate the flexibility of the proposed design in terms of the field penetration depth for a given coverage of the sample’s surface. Thus, at 282.6 MHz, we expected to reach a high penetration depth due to the first mode, while having a strong surface localization at 300.1 MHz due to the third mode. Provided that the aforementioned modes belong to different resonators, it is easy to tune their resonances to the Larmor frequencies almost independently by varying the lengths of the corresponding strips. Matching at both frequencies was achieved by adjusting their positions with respect to a common small loop feed connected to a 50-Ohm port. The resulting simulated frequency curve of the reflection coefficient *S*_11_ (dotted red line in Fig. 5(a)), shows that indeed dual-frequency tuning and matching is possible with the specific geometric parameters of the proposed coil ($|{S}_{11}|$ is much lower than −10 dB at both Larmor frequencies) with no lumped capacitors required. The magnetic field distributions created by the coil at these frequencies are presented in Fig. 6(a,c) and Fig. 6(b,d). As can be seen from the comparison of Fig. 6(b) and Fig. 6(d) with Fig. 2(d) and (g), the RF-field distributions obtained with the assembled coil are mostly determined by the desirable eigenmodes of the short-wire and long-wire resonators. However, a small field distortion at both frequencies can be observed in the location where the two resonators overlap (i.e. the location of the smaller short-wire resonator). The reason for this distortion is an inductive coupling between the selected eigenmodes of the resonators. The resonant frequency of each resonator (especially the frequency of the first-order mode) also becomes sensitive to tuning of the other resonator. Fortunately for the considered position of the two resonators, this sensitivity to the neighboring resonator is much weaker than the sensitivity to the individual tuning parameter of the coil, i.e. the length of the wires. Comparing the distributions inside the phantom at the two Larmor frequencies (Fig. 6(a,c)), it can be concluded that the field penetration at 282.6, due to Mode 1 of the short-wire resonator, is indeed deeper than that of the long-wire resonator’s Mode 3 at 300.1 MHz. This result is in qualitative agreement with the curves of Fig. 4. It should be noted that from the field maps in Fig. 6(a,c), one can clearly determine which resonator is responsible for excitation of the phantom at the Larmor frequencies. Therefore, as shown in the simulation, the proposed coil design allows tuning and impedance matching at two predefined Larmor frequencies using no lumped capacitive elements due to resonant excitation of the selected eigenmodes.

Despite the orthogonal orientation of the two resonators and the relatively small size of the short-wire resonator, the effect of their mutual coupling is still observable. Due to this effect and the influence of the RF-shield, the resonance frequencies of the selected modes moved from 309.9 MHz (Mode 3 of the long-wire resonator) and 264.4 MHz (Mode 1 of the short-wire resonator) to 300.1 and 282.6 MHz respectively. It should be noted that since the mutual coupling of the resonators is relatively weak, their resonant frequencies are mostly dependent on the length of their own strips.

By additional numerical simulation, we studied the combination where the first mode is excited in both the short-wire and the long-wire resonator. Simulation results show that inductive coupling between resonators is higher when the first mode is excited in both of them. This can be observed in Fig. 6, where the normal magnetic field component (e, j) and ${B}_{1}^{+}$ magnitude for 1 W of accepted power (f, h) of the modified coil is shown at 282.6 and 300.1 MHz. Despite both patterns being distorted due to the mutual coupling, the ${B}_{1}^{+}$ magnitude in the phantom at 300.1 MHz is higher when using the first mode of the long-wire than when using the third mode. Moreover, the distribution with the first mode is more uniform than with the third one. Significantly, since in the short-wire resonator in both compared cases we excited the same first mode, the ${B}_{1}^{+}$ magnitude and distribution at 282.6 MHz was hardly affected. This clearly demonstrates that with the proposed design one can select desirable operational eigenmodes in the two resonators of the coil.

The design, with Mode 1 of the short-wire and Mode 3 of the long-wire resonator, has been manufactured and tested on the bench and in MRI scans. The two *S*_11_ curves measured with a VNA on the bench (dashed blue curve in Fig. 5(a)) and using the MR system (solid green curve in Fig. 5(a)) demonstrate good agreement with the simulated data. The difference in *S*_11_ levels at the two resonances and the presence of additional parasitic resonances can be explained by the influence of a realistic MR-system’s bore geometry and the gradient system configuration, which was not considered in simulations. However, the experimental results confirm sufficient matching (${S}_{11} < -\,\,10$ dB at both the Larmor frequencies) required for dual-nuclei scanning.

The same coil setup with the phantom was used in the MRI scans. Comparing the obtained phantom images, one can conclude that the SNR decays with the depth in the phantom for ^1^H much faster than for ^19^F, which agrees with the numerically calculated field plots in Fig. 6(b,d). The SNR of the hydrogen image is also lower since the distance to the long-wire resonator from the phantom is slightly larger than the distance to the short-wire resonator. In fact, one could replace the long-wire and the short-wire resonators by each other to improve SNR in the top surface layer of the phantom for ^1^H. On the top left of the ^19^F image, a dark area can be observed. The origin of this effect is a high intensity of the ${B}_{1}^{+}$ in close proximity to the short-wire resonator, which is disposed just above the phantom. The flip angle in this part of the phantom becomes larger than the optimal one (close to 90 degrees) and is referred to as an overflip. The obtained phantom images clearly demonstrate that the field distributions created by the coil at two Larmor frequencies are almost similar in the axial direction of the MR-system, while the penetration depths are significantly different. This behavior of the coil results from the multi-mode nature of the periodic wire resonators and is determined by the selected eigenmode properties, as expected from simulations.

The obtained *in-vivo* images, presented in Figs 8 and 9, demonstrate that the designed coil is capable of ^1^H anatomic imaging of the top part of a body of a small animal with a wide coverage area in the coronal plane. The purpose of the obtained images is to demonstrate the capability of the coil to consequently give a signal at the two Larmor frequencies with a living subject without any adjustments. A quantitative comparison of the coil imaging capabilities to existing coils including *in-vivo* imaging is the subject of future work. As was previously shown, the limited ${B}_{1}^{+}$ penetration depth of the proposed coil at the Larmor frequency of ^1^H is due to the properties of the selected Mode 3 of the long-wire resonator. At the same time, the field of view of the coil is long in the *B*_0_ direction covering all the body length of a small animal, which also agrees with theoretical predictions. The coil was also capable of ^19^F imaging of the syringe under test using the same setup without retuning the coil. The performed dual-nuclei imaging has an appropriate quality for various biomedical applications of ^19^F/^1^H MRI.

## Methods

### Eigenmodes of wire metamaterial-inspired resonators

In order to numerically calculate the resonant frequencies of the two wire metamaterial-inspired resonators of the proposed coil (see Fig. 1(a)) and their eigenmode field distributions, we have made simulations using the Eigenmode Solver in CST Microwave Studio 2016 commercial software. For the short-wire and the long-wire resonators, the geometric parameters are illustrated in Fig. 2(a,b). Both resonators are single-layer flat periodic arrays of *N* = 6 thin printed copper strips of the width *w* = 1 mm and periodicity *a* = 10 mm. The substrate of the printed circuit board was 0.508-mm-thick Rogers 4003 laminate with permittivity *ε*_*r*_ = 3.38, and the dielectric loss tangent 0.0027. The long-wire resonator depicted in Fig. 2(a) has the length of strips *L*_1_ = 434.8 mm, which is comparable to one half of the wavelength at 300 MHz. The long-wire resonator was modeled alone in the lossless approximation, without a subject and a feeding loop. For the short-wire resonator, the same numerical eigenmode analysis was performed separately. The following geometric parameters of the short-wire resonator were set: *L*_2_ = 72 mm, *b* = 9 mm and *c* = 9.5 mm. All the patches from both ends of the strips of the short-wire resonator were printed on two separate Rogers 4003 grounded substrates of the thickness t = 0.508 mm (see Figs 1(a) and 2(b)). In the simulation, the number of analyzed modes was set to five for both resonators. The calculation domain was bounded by distant PEC walls from all sides. No phantom was included in the eigenmode simulation.

### Design and simulation of RF-coil

The whole proposed coil, assembled of two metamaterial-inspired resonators and a small loop feed, was simulated using Frequency Domain Solver in CST Microwave Studio 2016 together with a homogeneous phantom and a perfectly conducting cylindrical RF-shield of the diameter of 90 mm. The long-wire resonator was located on top of the short one as depicted in Fig. 1(a), so that the separation between their PCBs was 9 mm. A small inductively coupled feed was placed between the resonators and implemented as a flat annular copper ring of the width 4 mm and the external radius 20 mm, printed on 0.508-mm-thick Rogers 4003 C substrate. The loop was fixed 2 mm away from the plane of the long strips and 7 mm away from the plane of the short strips. As a result, a three-layer stack of PCBs was composed which operated as a dual-frequency surface coil. The length of the long-wire resonator and the mutual position of the latter and the feeding loop differs from the respective dimensions for mode 1–1 combination of the coil. Dimensions of the coil both for 1–3 and 1–1 combinations are listed in Table 2.

**Table 2:** Dimensions of the assembled coil for different mode combinations.

Parameter, mm	mode 1–3	mode 1–1
Long-wire resonator length, *L*_1_	434.8	449.8
Short-wire resonator length, *L*_2_	72	72
Long-wire resonator–feeding loop spacing	2	4
Short-wire resonator–feeding loop spacing	7	7

The coil produced an RF-field inside the tightly located cylindrical phantom with the diameter 40 mm, length 75 mm and material properties *ε*_r,phantom_ = 39 and *tanδ* = 0.06. In the simulation, the loop was driven by a lumped 50-Ohm port connected to its split, and the small loop itself excited one selected eigenmode in each of the two wire resonators. The parameters of the resonators were the same as previously used for eigenmode analysis. The values *L*_1_ and *L*_2_ were chosen in a parametric sweep performing multiple field simulation. The optimization goal was to achieve the calculated $|{S}_{11}|$ well below −10 dB at both considered Larmor frequencies. Impedance matching was reached by variation of the distances between the three aforementioned parallel PCBs of the RF-coil. Once tuning and matching was reached at the two desired Larmor frequencies, the circularly polarized RF field ${B}_{1}^{+}$ rotating in the axial plane with respect to the static field of the magnet corresponding to the accepted power of 0.5 W, was calculated using the template-based post-processing routine in the CST Microwave Studio. Sensitivity to loading variations of the proposed coil was studied through a number of simulations where the dimensions of the phantom were changed: radius of the phantom from the original value *R* = 20 mm to 0.8*R and 1.2*R and length of the phantom from *L* = 75 mm to 0.8*L and 1.2*L.

### On-bench measurements

**Figure 10:**
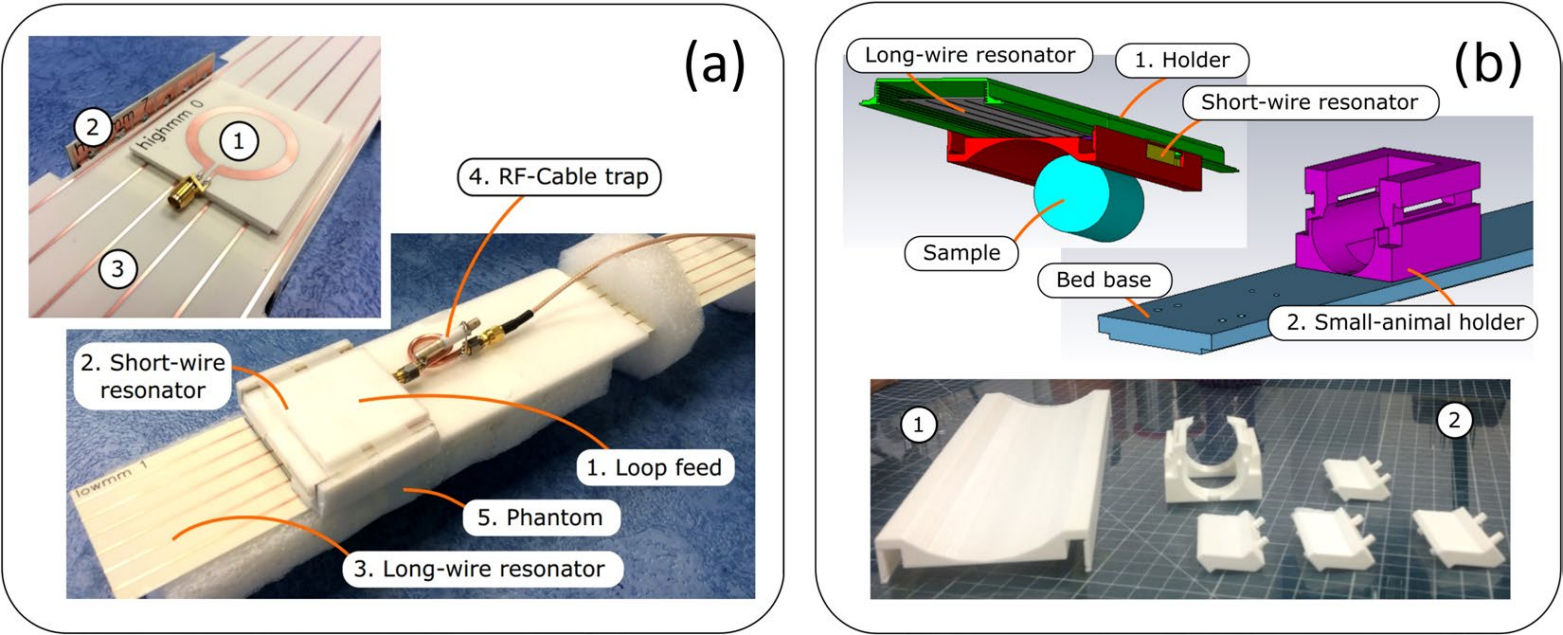
Experimental RF-coil: (**a**) PCB parts including loop feed, short-wire resonator and long-wire resonator and their assembly with spacer, phantom and RF-cable; (**b**) bed parts: holder for resonators and for small animal.

For on-bench and MRI experiments, the proposed coil was manufactured by manually assembling separate PCB parts, shown in the inset of Fig. 10(a), based on a common specially designed 3D-printed bed. The bed included a holder supporting the antenna parts shown in Fig. 10(a). The PCB parts in Fig. 10(a) are labeled as: 1–loop feed with SMA connector, 2–short-wire resonator with soldered PCBs supporting capacitive patches, 3–long-wire resonator. The bed also includes the base supporting the coil and a removable holder for a scanned sample, i.e. a small animal (see Fig. 10(b)). The whole bed fits the dimensions of a 90-mm diameter bore of a preclinical MRI. In Fig. 10(a), one can also observe the feeding coax with the RF-cable trap. The parts of the bed were manufactured by 3D printing.

All the geometry parameters of the two resonators in the manufactured coil correspond to ones chosen in the previously discussed simulation. The short-wire resonator was constructed of three 0.508-mm-thick Rogers 4003 PCBs: one supporting shortened copper strips and two grounded side PCBs with rectangular patches. All six strips from both ends were manually soldered to the corresponding patches printed on two-sided PCBs. The long-wire resonator was represented only by a single Rogers 4003 PCB with strips without connection to any capacitive loads. The coil was fed with a coaxial 2-mm 50-Ohm cable connected to the printed non-resonant circular loop through the cable trap with a cable ring shunted by a variable capacitor. The homogeneous phantom was represented by a cylindrical plastic can with the diameter 40 mm and height 75 mm filled with the solute of 60% 2-2-trifluorethanol and 40% water. The permittivity of 39 and the dielectric loss tangent 0.06 were measured for this liquid in the frequency range 100–400 MHz using the calibrated coaxial line section *EpsiMu* connected to the vector network analyzer (VNA) Anritsu MS2036C, and the obtained values were used in the performed numerical simulations.

In order to ensure proper tuning and impedance matching the reflection coefficient $|{S}_{11}|$ of the coil was measured first with the vector network analyzer (VNA) Anritsu MS2036C connected through a long calibrated cable to the coil located inside the bore of Bruker PharmaScan 7 T MR system. The result is shown in Fig. 5(a) with a dashed blue curve.

### MRI experiments with phantom and *in-vivo*

In order to validate the obtained tuning and impedance matching of the experimental coil, $|{S}_{11}|$ was again measured by means of the MR-system within its two operational bands (represented in Fig. 5(a) with the solid green curves).

The coil was tested by imaging of the phantom by scanning in the MR-system using the pulse sequence gradient echo (FLASH) TR/TE = 2000/2.4 ms with an isotropic voxel 0.7 × 0.7 × 0.7 mm^3^, the FOV was 44.8 × 44.8 mm, the number of averages was 1 and the flip angle was 90°. This flip angle was adjusted by Bruker reference power adjustment at the beginning of the scan in a horizontal slice located on the surface of the imaged sample.

*In-vivo* mouse acquisition was performed in accordance with European Union and French laws on animal experimentation (project authorization number: 12–058, site authorization number: B-91-272-01). The project authorization was delivered by the Ministry of Higher Education, Research and Innovation in France based on recommendations by the local ethical committee CETEA–CEA DSV IdF number 44. A mouse was placed under anesthesia (isoflurane 1-2%) in front of the center of the feeding loop. A 1 ml syringe filled with a mixture of 60% 2-2-trifluoroethanol and 40% water was attached to the back of the mouse as shown in Fig. 8(a). By scanning the syringe at the fluorine frequency, we aimed to demonstrate that the coil was capable of receiving the corresponding signal in the same configuration as was used for body imaging at the hydrogen frequency without injection of the fluorine-containing liquid. The mouse was positioned under the RF-coil’s resonators in front of the loop coil. Two sets of images were acquired sequentially at Larmor frequencies of 282.6 MHz for ^1^H and 300.1 MHz for ^19^F without the need for any intervention on the coil in between acquisitions. Fourteen transverse slices were acquired using a gradient echo sequence (FLASH) TR/TE = 2000/2 ms with a voxel size 0.5 × 0.5 mm^2^ and the slice thickness of 2 mm, the FOV was 32 × 32 mm, the number of averages was 1 and the flip angle was 90° (Fig. 9). In addition to transverse images, a coronal image at a higher resolution was also acquired by a gradient echo sequence (FLASH) TR/TE = 2000/4.6 ms with the spatial resolution of 0.25 × 0.25 mm^2^ and the slice thickness of 0.5 mm, the FOV was 80 × 32 mm, the number of averages was 1 and the flip angle was 90° (see the obtained whole-body image in Fig. 8(b)).

## Conclusion

In this work, a new design for a dual-nuclei preclinical RF-coil was proposed and characterized. The operational principle of the coil is based on resonant excitation of eigenmodes in a pair of multi-mode wire metamaterial-inspired resonators. It was numerically and experimentally shown that by proper selection of the excited eigenmodes, one can control the penetration depth into a subject, i.e. to efficiently excite the whole body or just a particular surface of a small animal. The resonant frequencies of the selected modes can be tuned by geometrical properties of the resonators of the coil, while impedance matching is determined by mutual positions of the resonators and the feeding annular loop. Proof-of-concept MRI tests on a phantom and *in-vivo* were performed showing imaging capability of the proposed coil at two Larmor frequencies of ^1^H and ^19^F at 7 Tesla. The proposed coil design is also compatible with multi-nuclei MRI applications using other nuclei (e.g. ^23^Na^31^,P etc.) since both the short and the long-wire resonators can be tuned to various Larmor frequencies by adjusting their length and the parameters of patches (providing the distributed load capacity for the short-wire resonator). Crucially, the proposed self-resonant design is cheap as it contains no variable non-magnetic capacitors for tuning and matching and can be constructed of just a few PCB parts which are movable against each other.
